# Prevalence, genetic diversity, and antimicrobial susceptibility of *Vibrio* spp. infected gilthead sea breams from coastal farms at Damietta, Egypt

**DOI:** 10.1186/s12917-024-03978-0

**Published:** 2024-04-01

**Authors:** Esraa Tawfeek Ismail, Mai A. M. El-Son, Fatma A. El-Gohary, Eman Zahran

**Affiliations:** 1 Department of Aquatic Animal Medicine, Faculty of Veterinary Medicine, Mansoura University, Mansoura, 35516 Egypt; 2 Department of Hygiene and Zoonoses, Faculty of Veterinary Medicine, Mansoura University, Mansoura, 35516 Egypt

**Keywords:** Mariculture, Bacterial outbreaks, PCR, ERIC, Antimicrobial susceptibility

## Abstract

**Background:**

Vibriosis is one of the most serious bacterial diseases and causes high morbidity and mortality among cultured sea breams*.* This study was undertaken to track the surveillance of *Vibrio* infection and its correlation to environmental factors*.* A total of 115 gilthead sea breams were collected seasonally from a private earthen pond fish farm in the Shatta area of Damietta, Egypt from September 2022 to July 2023. Physicochemical parameters of water were analyzed, and heavy metal levels were measured. The fish samples were subjected to clinical, bacteriological, Enterobacterial Repetitive Intergenic Consensus (ERIC) fingerprinting, and hematoxylin and Eosin histopathological staining.

**Results:**

The results revealed significant variations in the water quality parameters over different seasons, in addition to an increase in heavy metals. Naturally infected fish showed external signs and postmortem lesions that were relevant to bacterial infection. Two dominant *Vibrio* subspecies of bacteria were identified: *V. alginolyticus* (205 isolates) and *V. fluvialis* (87 isolates). PCR confirmed *the presence of V. alginolyticus* using the species-specific primer collagenase at 737 bp. The highest prevalence of *V. alginolyticus* was detected during the summer season (57.72%), and the lowest prevalence was observed in autumn (39.75%). The correlation analysis revealed a positive relationship between *V. alginolyticus* and water temperature (*r* = 0.69). On the other hand, *V. fluvialis* showed a high prevalence during the autumn season (25.30%) and the lowest prevalence during the summer season (10.56%), where it was negatively correlated with water temperatures (*r* =—0.03). ERIC fingerprinting showed genetic variation within the *Vibrio* isolates. Antimicrobial susceptibility testing revealed sensitivity to ciprofloxacin and doxycycline, and resistance to amoxicillin and erythromycin. The multiple antibiotic resistance (MAR) index values for *V. alginolyticus* and *V. fluvialis* ranged from 0.3 to 0.7, with a multi-drug resistance pattern to at least three antibiotics. Histopathological alterations in the affected tissues revealed marked hemorrhage, vascular congestion, and hemosiderosis infiltration.

**Conclusion:**

This study provides insights into the potential propagation of waterborne diseases and antibiotic resistance in the environment. Ensuring that the environment does not serve as a reservoir for virulent and contagious *Vibrio* species is a critical concern for regional aquaculture industries. Therefore, we recommend implementing environmental context-specific monitoring and surveillance tools for microbial resistance.

**Supplementary Information:**

The online version contains supplementary material available at 10.1186/s12917-024-03978-0.

## Introduction

In northern Egypt, particularly the regions of Damietta, Port Said, Alexandria, and the Suez Canal, mariculture is predominantly practiced, such as meagre (*Argyrosomus regius*), mullet (*Mugil cephalus*; *Liza ramada*), European sea bass (*Dicentrachus librax*), and gilthead sea bream (*Sparus auratus*). Marine aquaculture is an important sector contributing to the economic industry [1]. In Egypt, the mariculture sector is comparatively underdeveloped compared to the freshwater aquaculture industry; however, it shows significant growth contributed by several aquatic marine resources to overcome the scarcity and limitation of freshwater resources [2]. The semi-intensive aquaculture system is the most widely used, accounting for 80% of Egypt's total production. However, there has also been significant growth in intensive systems using tanks and cages [3, 4], and with the expansion of national marine initiatives, Egypt is currently among the top producers of gilthead sea bream [5, 6].

Climatic changes as a global concern influenced marine ecosystems [7], where these changes interact with anthropogenic effects like water management practices (e.g., drainage and pollution runoff) [8]. Higher temperature and salinity and lower pH and dissolved oxygen as climatic-driven change cascades would undoubtedly affect fish health [9], indeed infectious diseases, which respond acutely to climate changes [10, 11]. Infectious bacterial diseases rely on antimicrobials for treatment; however, the vast use of antimicrobials has resulted in bacterial resistance, rendering many known antimicrobials ineffective [12]. This phenomenon is a critical public health issue because of its direct correlation with the management and control of diseases [13]. As a result, it is critical to have up-to-date information on bacterial prevalence and species diversity, as climatic changes-driven factors led to bacterial adaptation with subsequent emergence of antibiotic resistance.

Vibriosis is ubiquitous in marine environments; they naturally exist apart from normal microbiota; however, pathogenic bacteria can also be isolated from healthy fish, pond sediment, and the prevalence of vibriosis is usually correlated to ecological and physico-chemical parameters of water [14]. *V. alginolyticus* and *V. fluvialis* are two important *Vibrio* species that are implicated in fish mortality and human illness [15]*. V. alginolyticus*, a prominent fish pathogen within the genus *Vibrio*, is the most prevalent and highly abundant species in marine environments and has been linked to numerous epizootic outbreaks involving gilthead sea bream and sea bass, causing severe infections and mortality [16, 17]. Infection with *V. alginolyticus* causes exophthalmia, wounds, septicemia, corneal cloudiness, and death in fish and shrimp [18]. *V. fluvialis* is usually isolated from marine mollusks, such as mussels and oysters, is considered one of the most emerging food pathogens worldwide, and commonly causes food poisoning [19, 20]. *V. fluvialis* has been occasionally isolated from cultured fish such as gilthead sea bream, European sea bass [21], and thin lip grey mullet *(Liza ramada*) [22], and is an emerging pathogen [15].

PCR-based methods are specific, quick and widely used to detect *Vibrio* spp., collagenase has been widely used as a biomarker for *V. alginolyticus* [23]. A number of opportunistic pathogens in fish and other marine animals are among the diverse range of vibrios and the intricate phylogeny of these organisms [24], indicating that vibriosis is a complex disease with multiple underlying causes. Many novel species of the genus that were previously impossible to distinguish using standard diagnostics have been discovered thanks to recent advancements in genomic science [25], in this context, ERIC-PCR has been used for detecting a genetic diversity between *Vibrio* species or within the same subspecies [26].

Therefore, the present study was undertaken to investigate the water quality parameters, heavy metals levels and seasonal prevalence of *Vibrio* spp infection in earthen ponds reared-sea bream during September 2022- July 2023, using both morphological and molecular techniques. Furthermore, this study explored the diversity of vibrios using ERIC and elucidated the antibiotic resistance patterns of the isolated strains.

## Material and methods

### Ethical committee consideration

All procedures and study protocols were reviewed and approved by the local guidance of the Research Ethics Committee of the Faculty of Veterinary Medicine, Mansoura University, Egypt (Code: M /111). All fish handling procedures and regulations followed the ARRIVE guidelines for Animal Care and Use.

### The study area

The current study was carried out in a period from September to 2022- July 2023, in a private fish farm located in the Shatta area at Damietta Governate, where the water source was from Manzala Lake. The farm visits were conducted seasonally on a bimonthly basis. A structured personally administered questionnaire was performed through an oral interview as described elsewhere with brief modification for gathering data about the farm managemental practice and fish history [27].

### Water sampling and assessment of water quality parameters

Water samples (100 mL) were collected seasonally in sterile 500-ml plastic bottles and stored according to standard methods described elsewhere [28, 29]. Temperature, PH, and Salinity were measured in situ at the farm using portable meters (Lovibond®, Dortmund, Germany), whereas heavy metals, including Iron, Mercury, Zinc, Nickel, Cadmium, Arsenic, and Lead were measured once per season using inductively coupled plasma (Thermo Scientific iCAP 7400) according to the methods adopted by Horwitz [29].

### Fish sampling and samples processing

A total of 115 gilthead seabreams, with an average weight of (25-50g), were collected seasonally. The collected fish samples were stored in isothermal boxes with an.

ice and immediately transported to the Aquatic Animal Medicine Laboratory (AAML), Faculty of Veterinary Medicine, Mansoura University. The collected fish were subjected to external and internal (postmortem) examinations, according to Austin and Austin [30] for any clinical abnormalities.

### Bacteriological isolation

Fish body surfaces were disinfected using 70% alcohol (Al-Goumhoria Co., Egypt) and aseptically dissected using a 3-cut incision. Loopful from kidney, liver, and brain were collected under complete aseptic conditions and inoculated into tryptic soy broth TSB (Oxoid, UK) supplemented with 1.5% NaCl, incubated at 28°C for 24–48 h. A loopful of the incubated broth was streaked onto thiosulfate citrate bile salt (TCBS) agar plates (Oxoid, UK). All plates were incubated at 28°C for 24–48 h, and colonies were purified by multiple re-streaking on TSA plates and stored in 30% glycerol (v/v) at ‒80°C.

### Biochemical identification

The obtained isolates were examined through assessment of morphological and phenotypic characteristics using motility, Gram’s stain, oxidase, and catalase tests followed by identification using DL D2mini Microbial ID & AST system** (**ZHUHAI DL BIOTECH Co., Ltd), according to the manufacturer’s instruction.

### Molecular identification of *V. alginolyticus*

#### Polymerase chain reaction (PCR) assay

*V. alginolyticus* isolates were selected for PCR confirmation as the one mostly obtained in the present study. The presumptive *V. alginolyticus* isolates based on biochemical identification were cultured in TSB for genomic DNA extraction. DNA extraction was performed using the boiling method described by Scarano et al. [31]. Briefly, the purified suspected colonies were sub-cultured in broth medium (TSB) and incubated at 28°C for 24 h. Next, one mL of broth culture was placed in an Eppendorf tube and centrifuged at 10,000 rpm for 5 min. The supernatant was discarded, 200 µL of Tris–EDTA (TE) buffer was added to the pellet, and the mixture was vortexed for dissolution. The Eppendorf tubes were boiled at 100 °C for 10 min and allowed to cool at ‒20 °C for 10 min, followed by centrifugation at 10,000 rpm for 10 min. The clear supernatant was ready for use as template DNA in the PCR assay. Then a species-specific primer, collagenase, was used to detect *V. alginolyticus* isolates F: (5-CGAGTACAGTCACTTGAAAGCC-3) R: (5 CACAACAGAACTCGCGTTACC-3) producing 737pb fragment [32]. The reaction mixture was performed in a total volume of 20.00 µL containing 10.00 µL master mix (SolGent™ 2X EF-Taq PCR Smart mix), 1.00 µL of each primer, 6.00 µL of nuclease free water, and 2.00 µL of DNA template. The PCR was performed using a thermocycler (Techne, UK), and the thermal conditions were as follows: initial denaturation at 95^◦^C for 2 min, followed by 35 cycles of denaturation at 95°C for 20 s, annealing at 57°C for 40 s, and extension at 72°C for 1 min; and finally, the final extension step at 72 °C for 5 min. The amplified products were allowed to migrate on 1% agarose gel for 45 min. Bands were visualized using a UV transilluminator (Spectroline, USA).

#### Enterobacterial Repetitive Intergenic Consensus (ERIC) sequences PCR (ERIC-PCR) and genetic fingerprinting analysis

ERIC analysis was performed to assess the clonal relationships among all obtained isolates using two primer sequences, ERIC1 (5’ ATGTAAGCTCCTGGGGATTCAC 3’) and ERIC2 (5′AAGTAAGTGACTGG GGTGAGCG 3′), as described previously (Siddique et al. 2021), where the extracted DNA from *Vibrio* isolates (*n* = 53) was used as a template for ERIC-PCR fingerprinting. The reaction components were Master mix (12.5µl) (SolGent™ 2X EF-Taq PCR Smart mix), 2 µl of ERIC1 and ERIC2, 6 µl template DNA and 2.5 µl nuclease free water. PCR was conducted in a DNA thermocycler (Techne, UK) using the following conditions: initial denaturation at 95°C for 5 min, followed by 35 cycles of denaturation at 95^◦^C for 45 s, annealing at 52^◦^C for 1 min, and extension at 72^◦^C for 3 min, followed by a final extension step for 10 min at 72^◦^C. The amplified products were allowed to migrate on 1.5% agarose gel for 60 min. Bands were visualized using a UV transilluminator (Spectroline, USA).

### Screening of antibiotic sensitivity to the identified *Vibrio spp,* and multiple antibiotic resistance (MAR) index value

The identified *Vibrio* spp, namely, *V*. *alginolyticus* and *V. fluvialis* were tested using the disc diffusion method according to the guidelines recommended by the Clinical and Laboratory Standards Institute (CLSI) [33]. The results were interpreted according to CLSI Clinical and Laboratory Standards [34, 35]. In contrast, doxycycline was recognized according to Enterobacteriaceae based on CLSI (M100) [36] and erythromycin based on the method described elsewhere [37]. The following antimicrobials were tested: chloramphenicol (C, 30μg), florfenicol (FFC, 30µg), doxycycline (DO, 30µg), amoxicillin (AX, 25µg), ciprofloxacin (CIP, 5µg), and erythromycin (E, 15µg, Oxoid). Pure cultures of the identified isolates were cultivated overnight in TSB at 28 °C; then, 100 µL of broth culture was spread onto Mueller–Hinton Agar (MHA) (Difco Detroit, MI, United States) using sterile glass rods, and antibiotic discs were applied carefully and incubated at 28°C for 24 h. The Multiple Antibiotic Resistance (MAR) index was calculated for isolates that exhibited resistance to more than two antibiotics according to the following formula: MAR index = a/b (a: number of antibiotics resistant to the isolates, b: total number of antibiotics to which the isolates were exposed).

### Histopathological examination

Tissue samples from the liver, kidney, and brain of infected fish were fixed in 10% buffered formalin, embedded in paraffin, sectioned at 5 mm, and stained with hematoxylin and eosin (H&E), according to Bancroft and Gamble [38]. Microscopically, histopathological alterations in H&E-stained tissue sections were examined at low and high magnification power.

### Statical analysis

The data were first checked for normality and homogeneity using Kolmogorov–Smirnov and Levene's tests, respectively. The data were then analyzed using a simple descriptive statistical frequency distribution and percentage analysis. The prevalence of bacterial isolates was calculated using the following formula: prevalence % = (no. of the specifically selected isolates/total No. of the bacterial isolates obtained) × 100. Using GraphPad® statistics package version 8.4.2. (GraphPad Software, Inc., USA**)**, differences among months, seasons, and organs were calculated using chi-square as *P* value < 0.05, which was considered significant. Ecological parameters were analyzed using one-way ANOVA. Parametric correlation analysis was carried out to establish the degree of correlation between *Vibrio* prevalences and water parameters (temperatures, and salinity), Pearson product-moment correlation coefficient r was considered significant at *p* < 0.05 (2-tailed).

## Results

### Criteria of farm facility and cultured fish

**Fig. 1:**
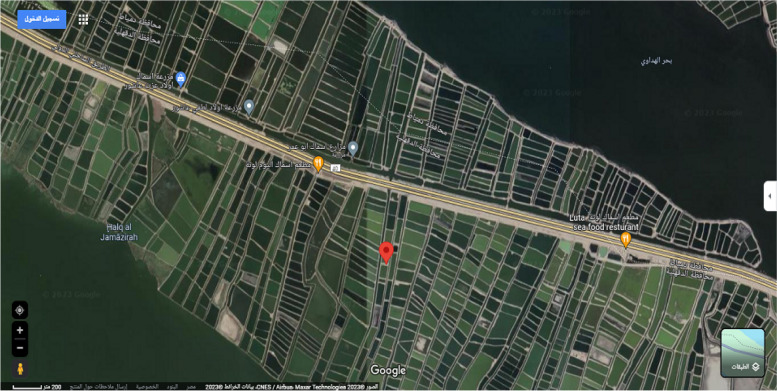
A map from google earth showing sampling site in seabream farm, Damietta, Egypt

The following information was gathered from a questionnaire (Table 1) evaluating the status of a fish farm managemental practices and fish history as well. The farm owner was 41 years old, had moderate education, and has been working on the farm for approximately 15 years. The farm is in the Shata area in Damietta province (Fig. 1) and spans an area of about 84000 m^2^. It includes several earthen ponds and mainly relies on semi-intensive production methods. The stocking density is about 300,000 fries per feddan, and the farm water supply is primarily from Lake El-Manzala. The farm uses feed supplements from a special feed store and allows for daily water exchange. Agrochemical products like disinfectants (hydrogen peroxide, H_2_O_2_) and veterinary drugs are only used when necessary for disease control. The farmer releases the fish when they reach a weight of about 50 g to continue growing in other farms. The farm produces an average of 50,000 juvenile fish per feddan, and according to the farm owner, there have been no recorded mortalities in the last two years, only sporadic cases.

**Table 1:** Questionnaire pertained to the farm managemental practices and the cultured fish

**Inquiry**	**Provided data**
**About owner**	
• **Age**	41
• **Gender**	Male
• **Education level**	Lower secondary
• **Experience level**	15 years
**About the farm**	
• **Farm location**	Shatta, Damietta
• **Type of production**	Semi-intensive
• **Farm size (feddan/m**^**2**^**)**	20 eddan
• **Production method**	Ponds
• **Fish stocking density/ feddan**	300.000
• **Water source of the farm**	Manzala lake
• **Water exchange rate/ pond**	Daily
• **Quality of pond water**	Turbid
• **Disinfectant used**	H2O2
• **Use of untreated poultry manure**	No
• **How dispose-off dead fish**	Burry
• **Have special food store (Yes/No)**	Yes
• **Veterinary supervision (Yes/NO)**	Yes
**About farmed fish (seabream)**	
• **Fish mortality rate**	Sporadic cases
• **Mortality rate in the last 2 years**	Not evident
• **Fish production rate**	50.000 fish
• **Marketing weight**	50 g

### Water quality parameters and heavy metals assessment

**Fig. 2:**
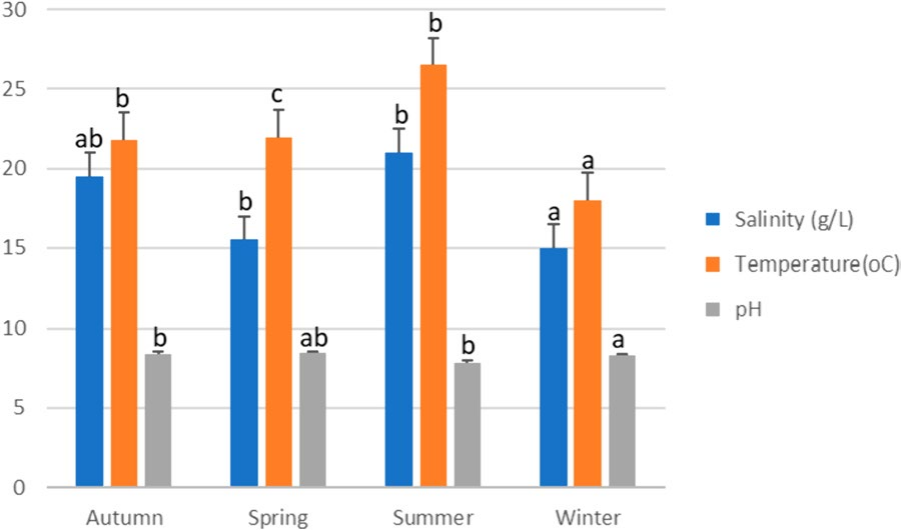
Water parameters measurements during different seasons in seabream farm. Data were represented as Mean ± SEM (*n* = 3). Values with a different letter superscript are significantly different between seasons (ANOVA with post hoc Tukey test, *P* < 0.05)

Water parameter measurements (Salinity, Temperature, and pH) were recorded during different seasons in the seabream farm, as shown in (Fig. 2). Salinity (g/L) showed a significant increase (*P* < 0.05) in summer (21 ± 0.01) compared to spring (15.5 ± 2.6) and winter (15 ± 1.73) without statistical changes compared to autumn (19.5 ± 0.29). Temperature (^o^C) was higher in summer (26.5 ± 0.2) than in spring (21.95 ± 0.61), autumn (21.75 ± 0.14) (*P* < 0.001), and winter (18 ± 1.04) (*P* < 0.0001). Meanwhile, pH values were lower in summer (7.85 ± 0.2) compared to spring (8.45 ± 0.2) and autumn (8.42 ± 0.05) (*P* < 0.05), with no significant between the latter. Seasonal concentrations of heavy metals (mg/L), including (Hg, Cd, Fe, Ph, Zn, As, and Cu) were higher than the permissible limit and are summarized in (Table 2).

**Table 2:** Heavy metals assessment (mg/L) in cultured seabream farm in different seasons

Heavy metals (mg/L)	Seasons				USEPA (2000)^a^	Law 48/1982^b^	FAO (**2017**)^c^
	**Autumn**	**Winter**	**Spring**	**Summer**			
**Hg**	0.196	2.56	5.35	0.28		0.001	
**Cd**	N. D	0.11	N. D	0.17	0.005	0.01	0.01
**Fe**	10.456	13.35	6.57	9.09	0.01	< 1	5
**Pb**	0.66	N. D	N. D	1.12	0.02	0.05	5
**Zn**	4.797	5.759	9.29	7.40	0.005	< 1	2
**As**	0.966	N. D	0.216	N. D	0.05	-	-
**Cu**	N. D	1.57	3.32	0.194	0.03	1	0.2

^a^USEPA (United states environmental protection agency) 2000. Risk-Based Concentration Table. United States Environmental Protection Agency, Philadelphia, PA, Washington, DC

^b^Law 48/1982: Egyptian Law for Protection of the River Nile and Waterways from Pollution, Art. (60) Water quality in the River Nile

^c^FAO: Food and Agriculture Organization Guidelines

### Clinical signs and postmortem lesion of gilthead seabream

**Fig. 3:**
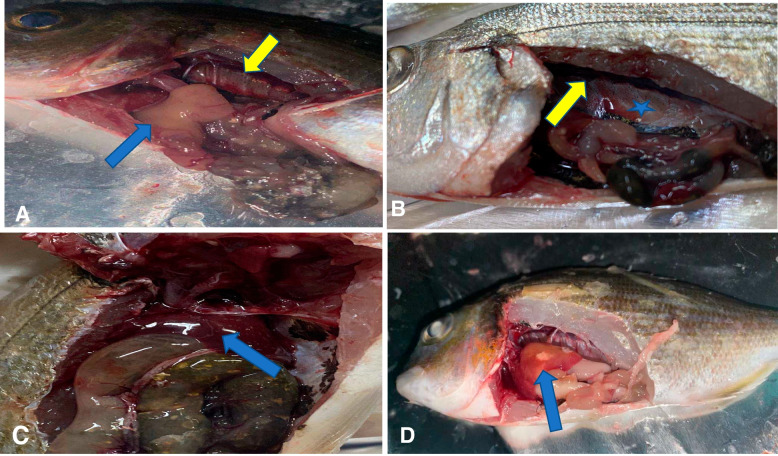
Naturally, infected seabream showing (**A**) pale anemic liver (blue arrow), congested kidney (yellow arrow). **B** Severely congested inflamed kidney (arrow) and pinpoint hemorrhage in the abdominal cavity. **C** severely congested liver. **D** presence of ischemic necrosis areas in the liver

The examined fish showed ocular opacity, fin erosion, and hemorrhagic spots on their body). Postmortem findings revealed congestion and enlargement of the kidney, brain hemorrhage, pale anemic liver with hemorrhagic patches, and marked visceral adhesions (Fig. 3).

### Bacteriological identification and biochemical characterization of isolates

A total isolates of 205 V. alginolyticus and 87 *V. fluvialis* were obtained. The *Vibrio* bacterial colonies were creamy white, round, and smooth on TSA medium. Presumptive *V. alginolyticus* bacterial colonies on TCBS agar plates appeared dark yellow round, large (2–4 mm), and mucoid, whereas *V. fluvialis* bacterial colonies appeared round, translucent yellow with shiny margins. Both *V*. *alginolyticus* and *V. fluvialis* were gram-negative, cytochrome oxidase-positive, catalase-positive, and reduced nitrate to nitrite. The purified colonies were further identified using the DL D2mini Microbial ID & AST system and confirmed as *V. alginolyticus* and *V. fluvialis* according to Bergey’s Manual (Table 3).

**Table 3:** Biochemical characterization of bacterial isolates retrieved from sea breams

**Criteria**	***V. alginolyticus***	***V. fluvialis***
**Colonies on TCBS**	Large dark yellow colony	Yellow colony with shiny margins *translucent*
**Gram stain**	Gram -ve straight to slightly curved rod	Gram -ve rods
**Motility**	Motile	Motile
**Oxidase**	+	+
**Catalase**	+	+
**Anerobic glucose fermentation**	+	+
**Hydrogen sulfide**	-	-
**Ornithine decarboxylase**	-	-
**Arginine bihydrolytic enzyme**	-	+
**Lysine decarboxylase**	+	-
**Urease**	-	-
**Citrate utilization**	-	-
**Nitrate**	+	+
**Indol**	+	-
**Mannitol acid production**	+	+
**Lactose acid production**	-	-
**Mannose acid production**	+	+
**Maltose acid production**	+	+
**Fructose acid production**	+	+
**Xylose acid production**	-	-
**β- galactosidase ONPG**	-	-
**Growth at 6.5% Nacl**	+	+

### Molecular identification and ERIC-PCR and genetic fingerprinting analysis

**Fig. 4:**
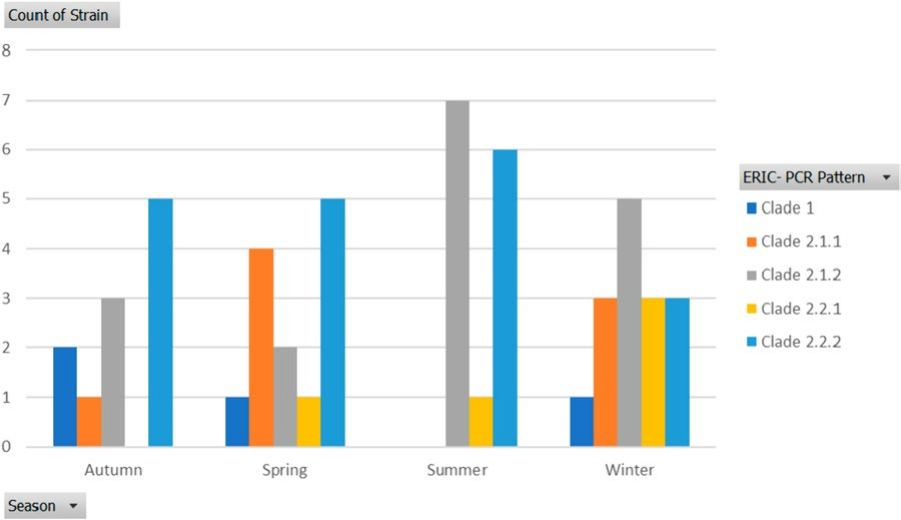
Seasonal representation of *Vibrio* isolates clades identified. Using PivotChart (Microsoft Excel, 2016) strains per clade were gathered in different seasons

**Fig. 5:**
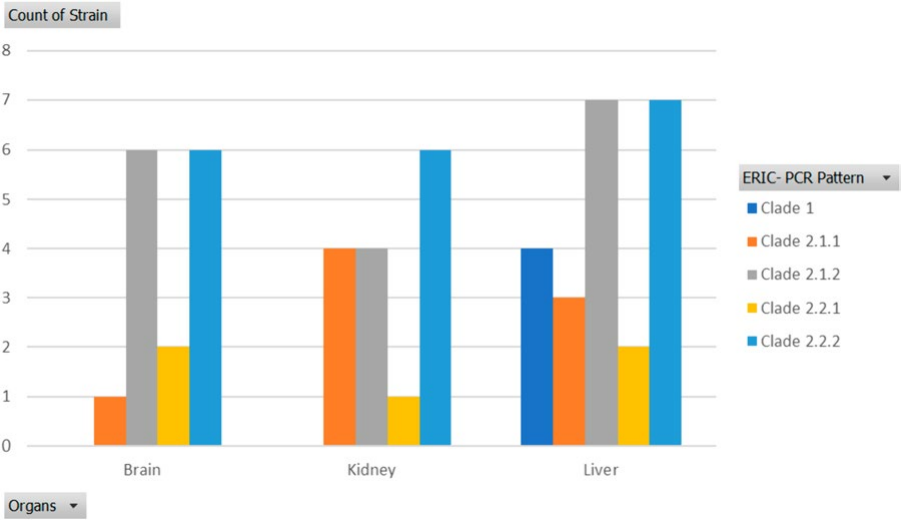
Organ’s representation of *Vibrio* isolates clades identified. Using PivotChart (Microsoft Excel, 2016) strains per clade were gathered in different organs

**Fig. 6:**
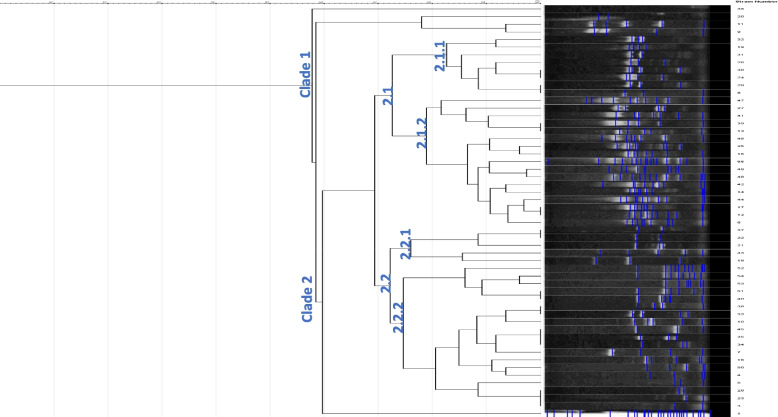
UPGMA cluster dendrogram of 53 pathogenic isolates based on molecular data generated from ERIC-PCR DNA fingerprint analysis

A total of 53 isolates of *V. alginolyticus* and *V. fluvialis* were selected out of all obtained isolates, based on representative number per each collection time. *V. alginolyticus* were identified using collagenase gene, species species-specific primers, results revealed 28.3% (15/53) of isolates were positive for the collagenase, positively amplified at 737 bp, and confirmed *V. alginolyticus* (Supplementary Fig. 1). The ERIC-PCR technique applied to 53 pathogenic isolates resulted in 2–16 amplification bands, with molecular sizes ranging from 150 to 3000 bp, where 150 bp was the common band for most isolates. Figures 4 and 5 demonstrated the isolate counts per clade across seasons and organs. All *Vibrio* isolates were divided into two major clades: clade 1 and clade 2. Clade 1 contained only four *Vibrio* strains, two of which (S9 and S11) were identified as *V. alginolyticus*. In contrast, the majority of other *Vibrio* isolates were grouped in Clade 2, which encompassed *V. alginolyticus* and *V. fluvialis*, in addition to other unidentified *Vibrio* spp. Data analysis was performed using GelJ software [39]. The Dice coefficient and unweighted-pair group method (UPGMA) generated a dendrogram (Fig. 6).

### Antibiotic sensitivity testing and MAR index value

Antibiotic susceptibility tests of *V. alginolyticus* and *V. fluvialis* (*n* = 22, 11 isolates each) were conducted against selected antibiotics belonging to different antimicrobial groups (Table 4). *V. alginolyticus* displayed 100% resistance to erythromycin and amoxicillin (Macrolides, Penicillin), followed 73% and 64% against chloramphenicol and florfenicol (Phenols); respectively. However, 100% of all isolates were sensitive to ciprofloxacin (quinolones) and doxycycline (tetracyclines). *V. fluvialis* isolates response against antibiotics were in the same pattern as *V. alginolyticus*, where they displayed 100% resistance to erythromycin and amoxicillin (Macrolides, Penicillin), followed 82% and 55% against chloramphenicol and florfenicol (phenols), respectively. However, 91% of isolates were sensitive to ciprofloxacin (quinolones) and doxycycline (tetracyclines). Table 5 shows that the MAR indices of the *V. alginolyticus* and *V. fluvialis* isolates ranged from 0.3 to 0.7. The MAR index values of all the bacterial isolates were higher than 0.2, indicating that most were from high-risk pollution sources. Moreover, the isolates showed multidrug resistance (MDR) patterns to at least three antibiotics.

**Table 4:** Antibiotic susceptibility of obtained *Vibrio* spp. to the selected antibiotics

		**V. *alginolyticus* (%) (*n* = 11)**			**V. *fluvialis* (%) (*n* = 11)**		
**Antimicrobial class**	**Antibiotics**	**S**	**I**	**R**	**S**	**I**	**R**
**Macrolides**	Erythromycin (E, 15 µg)	0 (0)	0 (0)	11 (100)	0 (0)	0 (0)	11 (100)
**Quinolones**	Ciprofloxacin (CIP, 5 µg)	11 (100)	0 (0)	0 (0)	10 (91)	1 (9)	0 (0)
**phenols**	Chloramphenicol (C, 30 µg)	0 (0)	4 (36.36)	7 (63.63)	0 (0)	2 (18.18)	9 (81.81)
	Florfenicol (FFC, 30 µg)	1 (9)	2 (18.18)	8 (72.72)	2 (18.18)	3 (27.27)	6 (54.54)
**Tetracyclines**	Doxycycline (DO-30 µg)	11 (100)	0 (0)	0 (0)	10 (91)	1 (9)	0 (0)
**Penicillin**	Amoxicillin (AX, 25 µg)	0 (0)	0 (0)	11 (100)	0 (0)	0 (0)	11 (100)

**Table 5:** Multiple antibiotic resistance indices and multiple drug resistance patterns of bacterial isolates

	**Antibiotics to which bacterial isolates showed resistance to**	**MDR pattern**	**MAR**
***V. alginolyticus isolates***			
S3, S18	AX, E	No	0.3
S4, S9, S10, S11, S17, S43	AX, E, FFC, C	Yes	0.7
S16, S49	AX, E, FFC	Yes	0.5
S55	AX, E, C	Yes	0.5
***V. fluvialis* isolates**			
S8, S42	AX, E	No	0.3
S33, S47	AX, E, C	Yes	0.5
S48	AX, E, C	Yes	0.5
S32, S45, S50, S51, S52	AX, E, FFC, C	Yes	0.7
S34	AX, E, FFC, C	Yes	0.7

### Prevalence of isolated *V. alginolyticus* and *V. fluvialis* in naturally diseased sea bream and its correlation to water parameters (temperatures and salinity)

**Fig. 7:**
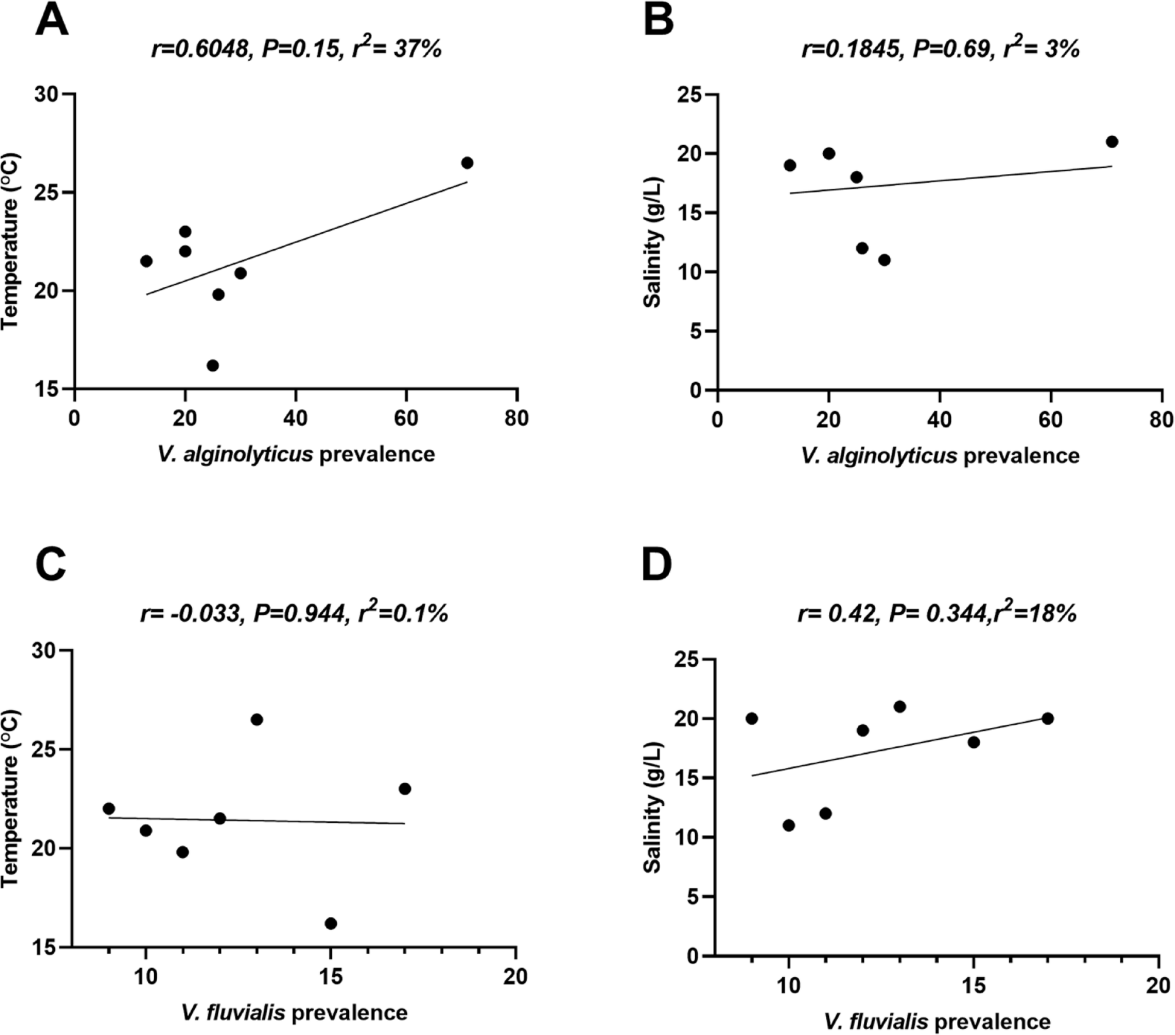
Comparative correlation profiles between *V. alginolyticus* (**A**, **B**), *V. fluvialis* (**C**, **D**), and water parameters. Results from water temperatures and salinity were correlated to *Vibrio* prevalence. Pearson correlation r coefficients are considered significant at *p < 0.05; **p < 0.01 (2-tailed)

The seasonal prevalence of *V. alginolyticus* and *V. fluvialis* showed significantly different seasons (Chi-square, *P* = 0.0075). Concerning *V. alginolyticus*, the prevalence was the highest in summer 57.72%, followed by winter 49.51%, spring 40.65%, and autumn 39.75%. While The prevalence of *V. fluvialis* was the highest in winter, followed by autumn, spring, and summer as follows 25.24%, 25.30%, 21.95%, 10.56%, respectively (Table 6). Similarly, monthly prevalence of *V. alginolyticus* and *V. fluvialis* showed significant differences across different months (Chi-square, *P* = 0.0053) (Table 7), where *V. alginolyticus* recorded the highest prevalence in July 57.7% and the lowest in November 29.54%). Meanwhile *V. fluvialis* exhibited the highest prevalence in February, 30%) and the lowest in July 10.5%). However, prevalences of both species were not significantly different among the organs (Chi-square, *P* = 0.3594) (Table 8). *V. alginolyticus* was the highest isolated from liver, followed by kidney and brain at 51.02%, 46.10%, 45.03%, respectively. Meanwhile, *V. fluvialis* prevalence was highly prevalent in brain, followed by kidney and liver as follows 23.66%, 20.12%, 17%, respectively (Table 8). Correlation analysis showed a non-significant positive relationship between *V. alginolyticus* prevalence and the water parameter (temperatures and salinity) (Fig. 7A,B). Additionally, the strength of the relationship between *V. alginolyticus* prevalence and water temperature was moderate (*r* = 0.6048), while week relationship had been noticed with salinity (*r* = 0.2). For *V. fluvialis*, correlation analysis showed a non-significant negative relationship between their prevalence and the water temperatures, while positive relationship with salinity (Fig. 7C,D). In addition, the strength of the relationship between *V. fluvialis* and water temperature was negligible (*r* =—0.03), while moderate relationship had been noticed with salinity (*r* = 0.42).

**Table 6:** Seasonal prevalence of *V. alginolyticus and V. fluvialis* isolated from sea breams

**Season**	**V. *alginolyticus***	**V. *fluvialis***	**chi-square *p*-value**
**Autumn**	39.75% (33/83)	25.30% (21/83)	0.0075
**Winter**	49.51% (51/103)	25.24% (26/103)	
**Spring**	40.65% (50/123)	21.95% (27/123)	
**Summer**	57.72% (71/123)	10.56% (13/123)

**Table 7:** Monthly prevalence of *V. alginolyticus and V. fluvialis* isolated from sea breams

**Month**	**V. *alginolyticus***	**V. *fluvialis***	**chi-square *p*-value**
**September**	51.20% (20/39)	23% (9/39)	0.0053
**November**	29.54% (13/44)	27.2% (12/44)	
**January**	49.05% (26/53)	20.75% (11/53)	
**February**	50% (25/50)	30% (15/50)	
**Mars**	46.15% (30/65)	15.3% (10/65)	
**May**	34.4% (20/58)	29.3% (17/58)	
**July**	57.7% (71/123)	10.5% (13/123)

**Table 8:** Prevalence of *V. alginolyticus and V. fluvialis* in different organs of sea breams

**Organs**	**V. *alginolyticus***	**V. *fluvialis***	**chi-square *p*-value**
**Liver**	51.02% (75/147)	17% (25/147)	0.3594
**Kidney**	46.10% (71/154)	20.12% (31/154)	
**Brain**	45.03% (59/131)	23.66% (31/131)

### Histopathological examination

**Fig. 8:**
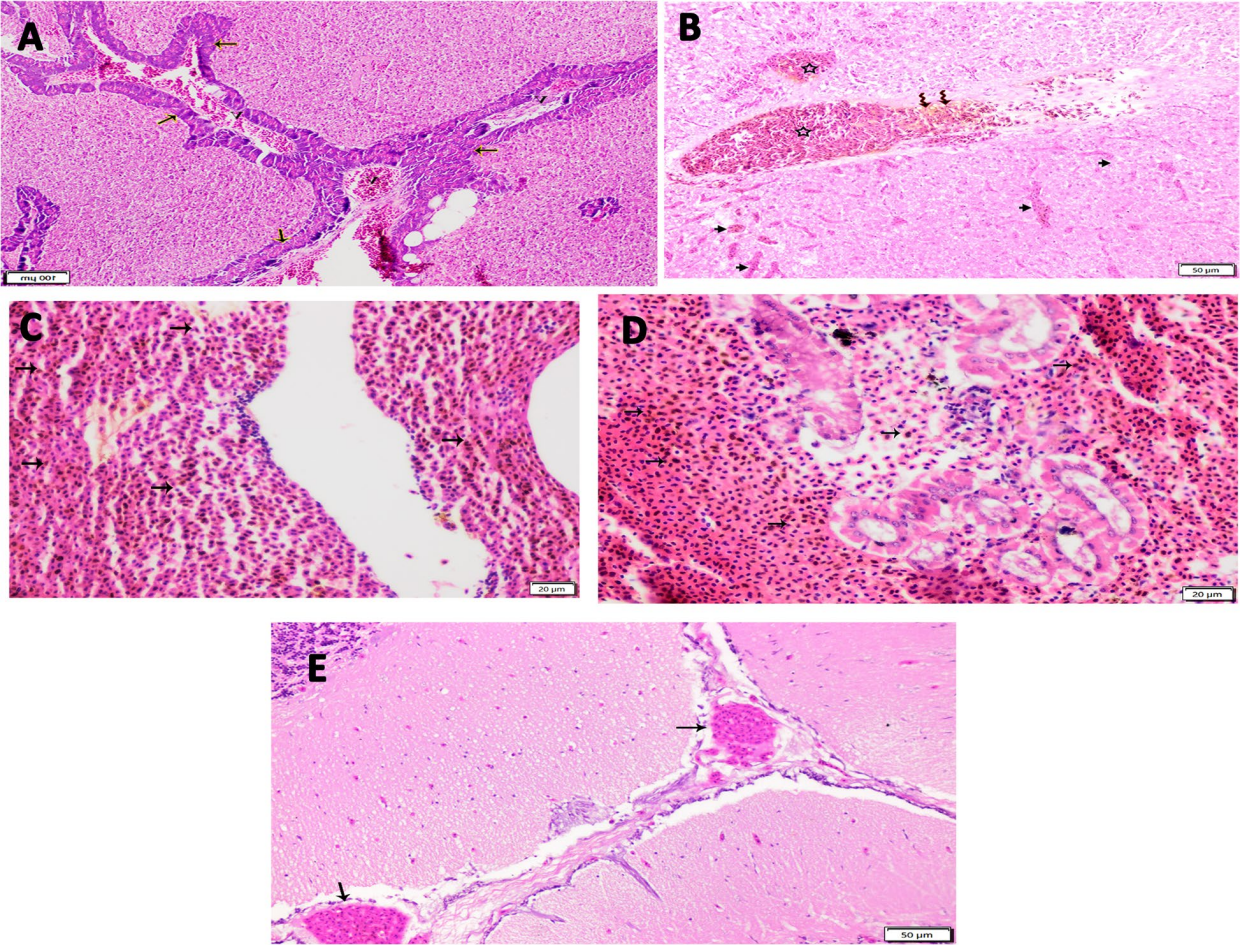
**A**-**E**. **A** Photomicrograph of a liver section from seabream infected with *Vibrio* sp. infection showed: severely congested veins in the hepatopancreatic tissue (V) and degenerated pancreatic acinar cells (arrows). 10X, H&E stain. **B** Higher magnification of a liver section from seabream with *Vibrio* sp. infection showed: severely congested veins (stars) containing hemosiderosis infiltrations (macrophages engulfed hemosiderin) (Zigzag arrows) and sever dilated and congested hepatic sinusoids (arrows). 40X, H&E stain. **C** Photomicrograph of a head kidney section from a seabream with *Vibrio* sp. infection showed: marked hemorrhage with diffuse hemosiderosis (macrophages engulfed hemosiderin) (arrows). 40X, H&E stain. **D** Photomicrograph of a trunk kidney section from a seabream with *Vibrio* sp. infection showed: marked hemorrhage with diffuse hemosiderosis (macrophages engulfed hemosiderin) (arrows). 40X, H&E stain. **E** Photomicrograph of the brain tissue section from a seabream with *Vibrio* sp. infection showed: severe vascular congestions (arrows). 10X, H&E stain

Histopathological examination of the liver revealed severely congested veins containing hemosiderosis infiltrations, and severely dilated and congested hepatic sinusoids (Fig. 8A, B). Both the head and trunk kidneys showed marked hemorrhage with diffuse hemosiderosis (Fig. 8C, D). The most characteristic finding in the brain tissue was severe vascular congestion (Fig. 8E).

## Discussion

Aquaculture is a vital global sector that contributes immensely to the supply of affordable animal protein to compensate for the global food shortage globally [40], and it is ranked as one of the most prevalent fish species among refined marine fish in Egypt. Most gilthead sea breams produced in North Egypt are in the triangular area between Damietta and Port Said Province [41].

Vibriosis is a potentially catastrophic bacterial disease that affects mariculture worldwide, causing high fatalities and significant economic losses [17]. In the present study, the clinical signs were slightly evident, unlike the postmortem lesions, which might be linked to the short fish production cycle in the examined farm, as the owner collected the fish at the juvenile stage (50 g) to complete the grow-out period on other farms. However, we could speculate that fish mortalities are evident in this farm since the owner stocked 300,000 fry/feddan and the production is 50,000 juvenile fish/feddan, which is a very low number related to what exactly be stocked. Similar to other studies, fish could serve as carriers and infect other susceptible fishes [14, 42]. In the present study, the obtained isolates were presumptively *Vibrio* spp. based on the morphological and biochemical identification, which were in full accordance with the standard reported in Bergey's manual of Systemic Bacteriology and matched to other studies [22, 43, 44]. As observed, juvenile fish (small-sized sea bream) demonstrated different prevalence of *Vibrio* infection, which is consistent with other studies reporting that small-sized fish with an ill-developed immune system have lower resistance to pathogens [45–47]; Another trail of studies reported that the incidence of *Vibrio* infection in farm-growing fish revealed the highest morbidity but a lower mortality rate [48, 49]. Postmortem findings herein aligned with previous studies [17, 50]. Additionally, the P.M lesion was evident histopathologically, where the liver and kidney showed severe congestion, hemolysis, and hemosiderosis (macrophages engulfed hemosiderin), confirming a subclinical *Vibrio* infection [51], which were in line with previous studies [50, 52, 53]. Postmortem lesions can be associated with pathogen invasion and release of extracellular metabolites, mainly bacterial hemolysins that cause lysis of erythrocytes and release intracellular hem; therefore, they are considered the main virulence factors that mostly contribute to signs of hemorrhagic septicemia. In addition, multiple virulent extracellular products, including proteases, lipases, siderophores, hyaluronidase, and collagenases, are associated with lesion development [54–56].

Recently, various molecular techniques have been adopted to determine genetic diversity, ERIC-PCR is one of these techniques that prove efficient, reliable, inexpensive, and simple to use [26]. In the present study, 15 of the 53 isolates were identified as *V. alginolyticus* using collagenase species-specific primers. Genotyping analysis using ERIC-PCR was carried out to elucidate the genetic diversity within all *Vibrio* spp. Isolates across different seasons within the fish farm. The ERIC-PCR pattern categorized all *Vibrio* isolates into two main clades (clade1, clade2), and most strains were clustered in clade 2, suggesting their genetic linkage, where they share the same source, and were collected within short time intervals. These findings are in line with previous reports [57–59].

The prevalence of *V. alginolyticus* in the current study was comparable with previous studies [58], reported high prevalence rate of *Vibrio* infected-sea bream in summer, with a prevalence rate of 82.61%, and the lowest was in autumn, with a prevalence rate of 30.23%. Similarly, Mahmoud et al. [60] recorded highest incidence of *V. alginolyticus* in sea bass during summer (63.33%) followed by winter (17.65%). These differences in prevalence percentages may be related to differences in area, fish immunity, fish species, water quality, and the number of fish sampled. The highest *V. alginolyticus* prevalence in summer followed by winter was believed to be associated with higher water temperatures (positive correlation, *r* = 0.69) and elevated levels of detected heavy metals, which diminish fish immunity and render them susceptible to infections [61–63]. Additionally, in our study, poor water quality and high levels of heavy metals, especially cadmium (Cd), lead (Pb), and nickel (Ni), during the summer season enhanced the susceptibility of fish to bacterial infections, explaining the high prevalence of the infection rate in summer [62, 64]. Furthermore, the highest iron level recorded in winter in this study was linked to the high prevalence of *V. alginolyticus*, suggesting a key role of iron level in enhancing *V. alginolyticus* infection. These findings concur with those of previous studies [63] that demonstrated a correlation between elevated levels of Cu, Fe, Ni, Cd, Co, Pb, and Cr in the water and the incidence of *Vibrio* infection, specifically *V*. *alginolyticus*, *V*. *harveyi*, *V*. *vulnificus*, and *V. mimicus.* On the other hand, *V. fluvialis* showed a high prevalence during all seasons except summer, evidenced by their negative correlation. While not assessed herein, a plausible explanation for this observation was given by Huang et al. [65], who reported that high temperatures led to the inactivation of type VI secretion systems (T6SSs), which are believed to enhance bacterial pathogenicity by exerting toxic effects on host cells or competing bacterial species; therefore, their prevalence rate was the lowest in summer.

In the current study, the *V. alginolyticus* load in the various organs of naturally infected seabream was in the order of liver > kidney > brain, which conformed with other investigations [58, 66, 67], reported that both the liver and kidney are the principal predilection organs for *V. alginolyticus*, while for *V. fluvialis* the pattern was brain > kidney > liver. According to the pathophysiological theory, the high prevalence in the liver and kidney is caused by some virulence factors acquired by invasive pathogens, which favor their existence in the primary detoxifying organ (liver) and immunological warrior (kidney) [68–70].

The antibiotic sensitivity and MDR pattern conform with those reported by El-Sayed et al. [71]. Antibiotic resistance indicates the presence of antibiotic residues in aquaculture ecosystems. In contrast, other variations in antibiotic sensitivity due to the greater development of antimicrobial resistance require the development of novel, potent antimicrobial drugs [72, 73]. When antibiotics are infrequently used at low doses, MAR values are typically equal to or less than 0.2 when antibiotics are used infrequently at low doses. In contrast, when the value is greater than 0.2, a high rate of antibiotic use in the treatment strategies is expected. In our study, the MAR index value was greater than 0.2, which indicates high-risk contaminated bacterial sources with high rates of antibiotic use [74, 75]. Most aquatic bacteria are subjected to many marine pollutants, such as agricultural runoff, sewage, and medical waste, which carry variable amounts of antimicrobials that trigger antimicrobial resistance in aquatic bacteria [76].

## Conclusion

Vibriosis is a serious fish pathogen often isolated from cultured sea breams. *V. alginolyticus* and *V. fluvialis* were most prevalent in summer and winter, respectively, and were associated with changes of water quality parameters and heavy metal levels. *Vibrio* spp. in this study, showed high variability in their genotypic traits, highlighting the importance of molecular identification as a diagnostic tool. Our findings emphasize the importance of accurately identifying *Vibrio* infections in aquaculture farms to monitor disease spread and occurrence, and objectively assess antimicrobial resistance profiles.

### Supplementary Information

**Additional file 1: Fig. 1.** Agarose gel electrophoresis of amplicons of positive V. alginolyticus isolates for collagenase gene 737bp, Lane M DNA ladder 100 bp, Lane 1,2,3,4 positive V. alginolyticus.

## Data Availability

All data supporting the findings of this study are available within the paper.

## References

[1] Shaalan M, El-Mahdy M, Saleh M, El-Matbouli M (2018). Aquaculture in Egypt: insights on the current trends and future perspectives for sustainable development. Rev Fisheries Sci Aquaculture.

[2] Sadek S (2000). Sea bream culture in Egypt; status, constraints and potential. Fish Physiol Biochem.

[3] Feidi I (2018). Will the new large-scale aquaculture projects make egypt self sufficient in fish supplies?. Mediterranean Fisheries Aquaculture Res.

[4] Kaleem O, Sabi AF (2021). Overview of aquaculture systems in Egypt and Nigeria, prospects, potentials, and constraints. Aquaculture Fisheries.

[5] Mehanna SF (2007). A preliminary assessment and management of gilthead bream* sparus aurata* in the port said fishery, the Southeastern Mediterranean Egypt. Turkish J Fisheries Aqua Sci.

[6] FAO (2020). FAO yearbook. Fishery and Aquaculture Statistics 2018/FAO annuaire.

[7] Danylchuk AJ, Griffin LP, Ahrens R, Allen MS, Boucek RE, Brownscombe JW, Casselberry GA, Danylchuk SC, Filous A, Goldberg TL (2023). Cascading effects of climate change on recreational marine flats fishes and fisheries. Environ Biol Fishes.

[8] He Q, Silliman BR (2019). Climate change, human impacts, and coastal ecosystems in the Anthropocene. Curr Biol.

[9] Little AG, Loughland I, Seebacher F (2020). What do warming waters mean for fish physiology and fisheries?. J Fish Biol.

[10] Aalto E, Lafferty KD, Sokolow S, Grewelle R, Ben-Horin T, Boch C, Raimondi P, Bograd S, Hazen E, Jacox M (2020). Models with environmental drivers offer a plausible mechanism for the rapid spread of infectious disease outbreaks in marine organisms. Sci Rep.

[11] Byers JE (2021). Marine parasites and disease in the era of global climate change. Ann Rev Mar Sci.

[12] Xu X, Cheng J, Wu Q, Zhang J, Xie T (2016). Prevalence, characterization, and antibiotic susceptibility of *Vibrio parahaemolyticus* isolated from retail aquatic products in North China. BMC Microbiol.

[13] Mo WY, Chen Z, Leung HM, Leung AO (2017). Research P: Application of veterinary antibiotics in China’s aquaculture industry and their potential human health risks. Environ Sci Pollut Res.

[14] Mohamad N, Mustafa M, Amal MNA, Saad MZ, Md Yasin IS, Al-saari N (2019). Environmental factors associated with the presence of Vibrionaceae in tropical cage-cultured marine fishes. J Aqua Anim Health.

[15] Arab S, Nalbone L, Giarratana F, Berbar A (2020). Occurrence of Vibrio spp. along the Algerian mediterranean coast in wild and farmed sparus aurata and dicentrarchus labrax. Veterinary World.

[16] Gobarah DEA, Helmy SM, Mahfouz NB, Fahmy HA, Abou MAEHM. Virulence genes and antibiotic resistance profile of *Vibrio* species isolated from fish in Egypt. In: Veterinary Research Forum: 2022. Urmia: Faculty of Veterinary Medicine, Urmia University; 2022. p. 315. 10.30466/vrf.2021.520767.3117 PMC9548226 36320310

[17] Abdelaziz M, Ibrahem MD, Ibrahim MA, Abu-Elala NM, Abdel-Moneam DA (2017). Monitoring of different *Vibrio* species affecting marine fishes in lake qarun and gulf of Suez: Phenotypic and molecular characterization. Egyptian J Aqua Res.

[18] Wang Y-D, Wang Y-H, Hui C-F, Chen J-Y (2016). Transcriptome analysis of the effect of Vibrio alginolyticus infection on the innate immunity-related TLR5-mediated induction of cytokines in Epinephelus lanceolatus. Fish Shellfish Immunol.

[19] Vinothkumar K, Bhardwaj AK, Ramamurthy T, Niyogi SK (2013). Disease I: Triplex PCR assay for the rapid identification of 3 major Vibrio species, Vibrio cholerae, Vibrio parahaemolyticus, and Vibrio fluvialis. Diagnostic Microbiol Infect Dis.

[20] Ramamurthy T, Chowdhury G, Pazhani GP, Shinoda S (2014). Vibrio fluvialis: an emerging human pathogen. Front Microbiol.

[21] Yiagnisis M, Athanassopoulou F (2011). Bacteria isolated from diseased wild and farmed marine fish in Greece. Recent Adv Fish Farms.

[22] Zaki VH, Gala AM, Eissa A (2018). COMMON VIBRIOS AFFECTING THINLIP GREY MULLET (LIZA RAMADA). JEVMA..

[23] Najwaa MN, Danielb AMD, Amin K, Effendya A (2015). Detection of virulence genes in Vibrio alginolyticus isolated from green mussel. Perna viridis J Teknol.

[24] Culot A, Grosset N, Bruey Q, Auzou M, Giard J-C, Favard B, Wakatsuki A, Baron S, Frouel S, Techer C (2021). Isolation of Harveyi clade Vibrio spp. collected in aquaculture farms: How can the identification issue be addressed?. J Microbiol Methods.

[25] Triga A, Smyrli M, Katharios P (2023). Pathogenic and opportunistic vibrio spp. Associated with vibriosis incidences in the greek aquaculture: the role of vibrio harveyi as the principal cause of vibriosis. Microorganisms.

[26] Cristiani M, Flores MJ, Brandi RJ, Tedeschi FA, Zalazar FE, Labas MD (2020). Biology PB: ERIC-PCR technique applied to monitoring and quantification of DNA damage during water disinfection process. J Photochemist Photobiol B.

[27] El-Gohary FA, Zahran E, Abd El-Gawad EA, El-Gohary AH, M. Abdelhamid F, El-Mleeh A, Elmahallawy EK, Elsayed MM: Investigation of the Prevalence, Virulence Genes, and Antibiogram of Motile Aeromonads Isolated from Nile Tilapia Fish Farms in Egypt and Assessment of their Water Quality. Animals 2020, 10(8):1432. 10.3390/ani10081432 PMC7459692 32824393

[28] Boyd CE (1990). Water quality in ponds for aquaculture.

[29] Horwitz W: Standard methods for the examination of water and wastewater. In.: APHA, Washington, DC; 2000.

[30] Austin B, Austin DA: Bacterial Fish Pathogens: Disease of Farmed and Wild Fish: Springer International Publishing; 2016.

[31] Scarano C, Spanu C, Ziino G, Pedonese F, Dalmasso A, Spanu V, Virdis S, De Santis EJ (2014). Antibiotic resistance of Vibrio species isolated from Sparus aurata reared in Italian mariculture. New Microbiol.

[32] Di Pinto A, Ciccarese G, Tantillo G, Catalano D, Forte VT (2005). A collagenase-targeted multiplex PCR assay for identification of Vibrio alginolyticus, Vibrio cholerae, and Vibrio parahaemolyticus. J Food Prot.

[33] Patel JB: Performance standards for antimicrobial susceptibility testing; twenty-fifth informational supplement. In.: Clinical and Laboratory Standards Institute; 2015.

[34] CLSI: Methods for dilution antimicrobial susceptibility tests for bacteria that grow aerobically. Approved Standard, Pennsylvania 2012:19087–11898.

[35] Jorgensen JHJ (2010). Methods for antimicrobial dilution and disk susceptibility testing of infrequently isolated or fastidious bacteria; approved guideline.

[36] CLSI (2018). Performance standards for antimicrobial susceptibility testing: 28th informational supplement: Clinical and Laboratory Standards Institute, Wayne, PA2018.

[37] Bauer AW, Kirby WM, Sherris JC, Turck M (1966). Antibiotic susceptibility testing by a standardized simple disk method. Am. J. Clin. Pathol..

[38] Bancroft JD, Gamble M: Theory and practice of histological techniques: Elsevier health sciences; 2008.

[39] Heras J, Domínguez C, Mata E, Pascual V, Lozano C, Torres C, Zarazaga M (2015). GelJ–a tool for analyzing DNA fingerprint gel images. BMC Bioinform.

[40] Soliman NF, Yacout DM (2016). Aquaculture in Egypt: status, constraints and potentials. Aquaculture Int.

[41] Megahed ME, Aly SM: Challenges facing marine aquaculture and requirements for development in egypt. In: Proceedings of the 2nd Global Fisheries and Aquaculture Research Conference, Cairo International Convention Center: 2009; 2009: 24–26.

[42] Regev Y, Davidovich N, Berzak R, Lau SC, Scheinin AP, Tchernov D, Morick DJ (2020). Molecular identification and characterization of vibrio species and mycobacterium species in wild and cultured marine fish from the Eastern Mediterranean Sea. Microorganisms.

[43] Abdelazeem E, Mabrok M, Megahed A, El-Lamie M (2023). Bacteriological studies on vibriosis in Egyptian Sole (Solea aegyptiaca) Fish. Suez Canal Veter Med J.

[44] Abdel-Aziz M, Eissa AE, Hanna M, Abou Okada M (2013). Medicine: Identifying some pathogenic Vibrio/Photobacterium species during mass mortalities of cultured Gilthead seabream (Sparus aurata) and European seabass (Dicentrarchus labrax) from some Egyptian coastal provinces. Int J Veterinary Sci Med.

[45] Abdullah A, Ramli R, Ridzuan MSM, Murni M, Hashim S, Sudirwan F, Abdullah SZ, Mansor NN, Amira S, Saad MZ (2017). The presence of Vibrionaceae, Betanodavirus and Iridovirus in marine cage-cultured fish: role of fish size, water physicochemical parameters and relationships among the pathogens. Aquaculture Rep.

[46] Bellos G, Angelidis P, Miliou H (2015). Effect of temperature and seasonality principal epizootiological risk factor on vibriosis and photobacteriosis outbreaks for european sea bass in greece (1998-2013). J Aquac Res Dev.

[47] Toranzo AE, Magariños B, Romalde JL (2005). A review of the main bacterial fish diseases in mariculture systems. Aquaculture.

[48] Mohi M, Kuratani M, Miyazaki T, Yoshida T (2010). Histopathological studies on Vibrio harveyi–infected tiger puffer, Takifugu rubripes (Temminck et Schlegel), cultured in Japan. J Fish Dis.

[49] Sumithra T, Reshma K, Anusree V, Sayooj P, Sharma S, Suja G, Amala P, Joseph S, Sanil NJ (2019). Pathological investigations of Vibrio vulnificus infection in genetically improved farmed tilapia (Oreochromis niloticus L.) cultured at a floating cage farm of India. Aquaculture.

[50] Aly S, Eisa A, ElBanna N (2019). Characterization of vibrio alginolyticus infection in gilthead seabream (Sparus Auratus, L) cultured in Egypt. Suez Canal Veterinary Med J.

[51] Ganesan S, Baskaran B, Raj M, Mandal A, Shanmugam K, Subramanian P, Tabarsa M, You SG, Narayanasamy Marimuthu PJTAIJoMS: Vibriosis Incidents in Marine Finfish Farms: Prevalence, Diagnosis of Pathogens using 16S rRNA, Histopathology, and In Vitro Antibacterial Evaluation Against Isolated Vibrio spp using Antibiotics and Probiotics. 2021:1–15.

[52] Manchanayake T, Salleh A, Amal MNA, Yasin ISM, Zamri-Saad M (2023). Pathology and pathogenesis of Vibrio infection in fish: a review. Aquaculture Rep.

[53] Eissa I, Aly S, Derwa H, Fawzy A (2017). Studies on vibriosis among some marine fishes in Lake Temsah. Suez Canal Veterinary Med J.

[54] Salamone M, Nicosia A, Ghersi G, Tagliavia M (2019). Vibrio proteases for biomedical applications: modulating the proteolytic secretome of V. alginolyticus and V. parahaemolyticus for improved enzymes production. Microorganisms.

[55] Zhang XH, Austin B (2005). Haemolysins in Vibrio species. J Appl Microbiol.

[56] Bunpa S, Sermwittayawong N, Vuddhakul V (2016). Extracellular enzymes produced by Vibrio alginolyticus isolated from environments and diseased aquatic animals. Procedia Chemist.

[57] Di Pinto A, Ciccarese G, Tantillo G, Catalano D (2005). Forte VTJJoFP: A collagenase-targeted multiplex PCR assay for identification of *Vibrio alginolyticus, Vibrio cholerae, and Vibrio parahaemolyticus*. J Food Prot..

[58] Abd El Tawab A, Ibrahim AM, Sittien A (2018). Phenotypic and genotypic characterization of Vibrio species isolated from marine fishes. Benha Veterinary Med J.

[59] Xie T, Yu Q, Tang X, Zhao J, He X (2020). Prevalence, antibiotic susceptibility and characterization of Vibrio parahaemolyticus isolates in China. FEMS Microbiol Letters.

[60] Mahmoud S, El-Bouhy Z, Hassanin M, Fadel AH (2018). Chemical, Sciences B: Vibrio alginolyticus and Photobacterium damselae subsp. Damsel: prevalence, histopathology and treatment in sea bass dicentrarchus labrax. J Pharmaceut Chemical Biol Sci.

[61] Lawson TJAITPC, USA: Fundamentals of Aquacultural Engineering. Chapman-Hall. 1995.

[62] Richards JG: Metabolic and molecular responses of fish to hypoxia. In: Fish physiology. Volume 27, edn.: Elsevier; 2009: 443–485.

[63] Elgendy M, Soliman W, Hassan H, Kenawy A, Liala AM (2015). Science A: effect of abrupt environmental deterioration on the eruption of vibriosis in mari-cultured shrimp, Penaeus indicus, in Egypt. J Fish Aquatic Sci.

[64] El-Son MA, Nofal MI, Abdel-Latif HM (2021). Co-infection of Aeromonas hydrophila and Vibrio parahaemolyticus isolated from diseased farmed striped mullet (Mugil cephalus) in Manzala, Egypt–a case report. Aquaculture.

[65] Huang Y, Du P, Zhao M, Liu W, Du Y, Diao B, Li J, Kan B, Liang W (2017). Functional characterization and conditional regulation of the type VI secretion system in Vibrio fluvialis. Front Microbiol.

[66] Ezzat M, Mohaeed G, Ad El-Hak M, Wahdan A (2018). Prevalence and genotypic characterization of vibrio alginolyticusin somemarine fishes. Suez Canal Vet Med J.

[67] El-Sayed M, Algammal A, Abouel-Atta M, Mabrok M, Emam AJ (2019). Pathogenicity, genetic typing, and antibiotic sensitivity of Vibrio alginolyticus isolated from Oreochromis niloticus and Tilapia zillii. Rev Med Vet.

[68] Botella S, Pujalte MJ, Macián MC, Ferrús MA, Hernández J, Garay E (2002). Amplified fragment length polymorphism (AFLP) and biochemical typing of Photobacterium damselae subsp. damselae. J Appl Microbiol.

[69] Zorrilla I, Chabrillón M, Arijo S, Dıaz-Rosales P, Martınez-Manzanares E, Balebona M, Morinigo MA (2003). Bacteria recovered from diseased cultured gilthead sea bream (Sparus aurata L.) in southwestern Spain. Aquaculture.

[70] Eissa I, Derwa H, El-Lamei M, Desuki A, Zaki M, El-Sheshtawy H (2013). Iron in water and some marine fishes in relation to vibriosis at Lake Temsah. Life Sci J.

[71] El-Sayed MR, Osman AE, Emam AM, El-Galil A, Sayed HH (2021). Studies on vibrio alginolyticus infection among some red sea fishes at Hurghada. Assiut Veterinary Med J.

[72] Abdallah EM, Abdalla WE (2018). Black pepper fruit (Piper nigrum L.) as antibacterial agent: a mini-review. J Bacteriol Mycol Open Access.

[73] Siddique AB, Moniruzzaman M, Ali S, Dewan MN, Islam MR, Islam MS, Amin MB, Mondal D, Parvez AK, Mahmud ZH (2021). Characterization of pathogenic Vibrio parahaemolyticus isolated from fish aquaculture of the southwest coastal area of Bangladesh. Front Microbiol.

[74] Salem M, Zharan E, Saad R, Zaki V (2020). Prevalence, molecular Cاharacterization, virulotyping, and antibiotic resistance of motile aeromonads isolated from Nile tilapia farms at northern Egypt. Mansoura Veterinary Med J.

[75] Krumperman PH (1983). microbiology e: multiple antibiotic resistance indexing of Escherichia coli to identify high-risk sources of fecal contamination of foods. Appl Environ Microbiol.

[76] Elgendy MY, Ali SE, Abbas WT, Algammal AM, Abdelsalam M (2023). The role of marine pollution on the emergence of fish bacterial diseases. Chemosphere.

