# Supplementary material for: Prevalence, genetic diversity, and antimicrobial susceptibility of Vibrio spp. infected gilthead sea breams from coastal farms at Damietta, Egypt
Source: BMC Vet Res. 2024 Apr 1;20:129. doi: 10.1186/s12917-024-03978-0 (PMC10986055; doi:10.1186/s12917-024-03978-0)
Supplement: Supplementary file 1 — Additional file 1: Fig. 1. Agarose gel electrophoresis of amplicons of positive V. alginolyticus isolates for collagenase gene 737bp, Lane M DNA ladder 100 bp, Lane 1,2,3,4 positive V. alginolyticus. [file 12917_2024_3978_MOESM1_ESM.pptx]

## Slide 1
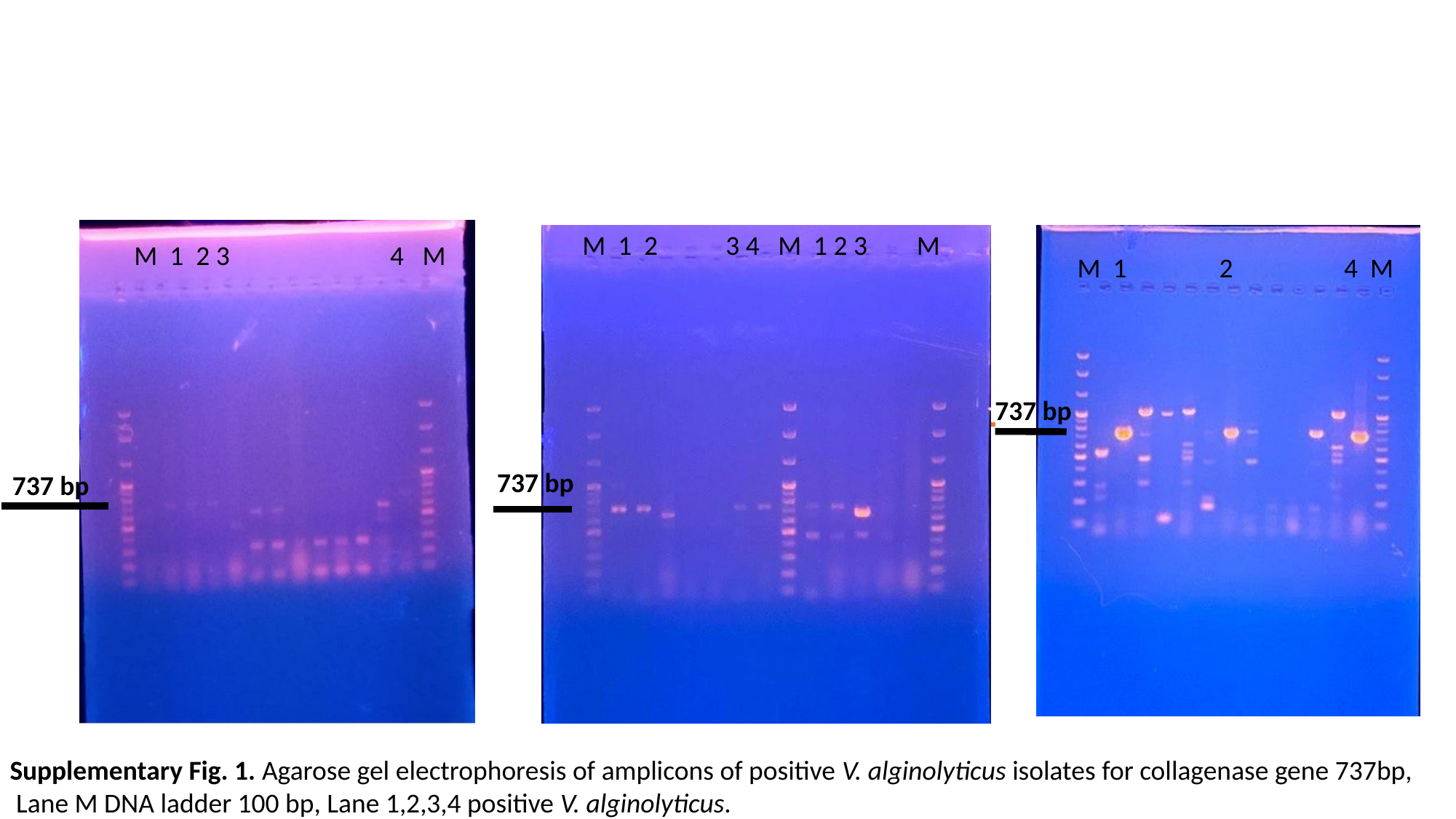

M 1 2 3 4 M 1 2 3 M
M 1 2 3 4 M
M 1 2 4 M
737 bp
737 bp
737 bp
737 bp
Supplementary Fig. 1. Agarose gel electrophoresis of amplicons of positive V. alginolyticus isolates for collagenase gene 737bp,
 Lane M DNA ladder 100 bp, Lane 1,2,3,4 positive V. alginolyticus.
